# The Changing Landscape of Sodium Needs in the Preterm Neonate for Optimizing Growth and Development

**DOI:** 10.3390/nu18020186

**Published:** 2026-01-06

**Authors:** Chrysoula Kosmeri, Maria Baltogianni, Niki Dermitzaki, Chrysanthi Maria Tsiogka, Vasileios Giapros

**Affiliations:** 1 Department of Pediatrics, University Hospital of Ioannina, 45500 Ioannina, Greece; chrisa.kosmeri@gmail.com; 2 Neonatal Intensive Care Unit, School of Medicine, University of Ioannina, 45500 Ioannina, Greece; mbalt@doctors.org.uk (M.B.); nikidermitzaki@yahoo.com (N.D.); chrisatsiogka@gmail.com (C.M.T.)

**Keywords:** sodium, hyponatremia, individualized supplementation, preterm neonate, growth, urinary sodium monitoring

## Abstract

**Sodium** (Na) is essential not only for maintaining extracellular fluid homeostasis as the dominant extracellular cation, but also for supporting the rapid tissue growth characteristic of the neonatal period. Despite its importance, the precise sodium requirements of preterm infants remain insufficiently defined. The immature renal tubules of preterm neonates lead to significant renal sodium losses, making negative sodium balance a common feature in this population. This issue has become increasingly relevant as survival rates improve among extremely preterm infants, while most available data are derived from studies involving more mature preterm or even full-term neonates. Fractional excretion of sodium (FENa) shows a clear inverse correlation with both gestational age and postnatal age, highlighting the developmental limitations in sodium retention among the youngest and most vulnerable infants. Current guidelines on sodium supplementation aim to promote optimal growth and neurodevelopment but vary across organizations. For instance, the most recent ESPGHAN recommendations suggest higher sodium intakes, in the range of 3–8 mEq/kg/day, whereas the American Academy of Pediatrics (AAP) provides more conservative guidance. These discrepancies underscore ongoing uncertainty in determining optimal sodium provision. This narrative review examines both classic and contemporary data on sodium needs in preterm neonates, with the goal of clarifying existing evidence and offering practical insights for clinical care. It also emphasizes unresolved questions and the need for well-designed studies that address the unique physiology of extremely preterm infants. A deeper understanding of sodium metabolism in this population is crucial for improving outcomes and guiding evidence-based supplementation strategies.

## 1. Introduction

Sodium (Na), together with its accompanying anions, is the primary determinant of extracellular fluid osmolality, accounting for nearly 90% of it. By shaping the osmotic balance between body compartments, sodium plays a central role in regulating fluid distribution, blood pressure, and the electrical activity of nerve and muscle cells. Although serum sodium reflects the osmotic component of total body sodium, it does not capture sodium that is stored in non-exchangeable tissue compartment ς, an aspect of sodium distribution that remains quantitatively unclear [1].

Maintaining total body sodium within a narrow range is essential for physiological stability [2]. Sodium homeostasis is primarily achieved by the kidneys, which regulate sodium reabsorption and excretion along different nephron segments. Sodium is freely filtered at the glomerulus, and approximately 65–70% of the filtered amount is reabsorbed in the proximal tubule. Further reabsorption occurs in the loop of Henle, distal convoluted tubule, and collecting ducts. This process is under hormonal and neural control, mainly involving the renin–angiotensin–aldosterone system, antidiuretic hormone, natriuretic peptides, and the sympathetic nervous system [2,3].

Preterm infants are particularly susceptible to dysnatremia, with early hypernatremia and later hyponatremia being the most frequent disturbances [4]. Hyponatremia in the first 4–5 days of life is typically due to excess total body water rather than sodium loss, most often from overhydration or, less commonly, syndrome of inappropriate antidiuretic hormone [5,6]. Beyond this period, hyponatremia usually reflects true sodium depletion resulting from immature tubular function and inadequate replacement of ongoing renal sodium losses [7]. Hypernatremia generally arises from excessive water loss or insufficient fluid replacement, often presenting as excessive postnatal weight loss. When hypernatremia occurs without dehydration, iatrogenic sodium overload (from parenteral fluids, medications, or transfusions) should be considered [7,8].

Sodium balance is closely linked to growth and neurodevelopment in preterm infants. Without adequate supplementation, persistent renal sodium losses often lead to negative sodium balance, growth restriction, and potentially adverse neurodevelopmental outcomes [4,9]. Optimal intake of macronutrients and sodium in the first postnatal month is therefore crucial to support weight gain and brain development, particularly in very preterm neonates [10,11,12,13]. Even with modern nutritional strategies, nearly 50% of very-low-birth weight infants still experience postnatal growth failure, a problem likely influenced by sodium depletion secondary to renal losses [14]. Current international guidelines vary regarding the recommended sodium intake for preterm infants. The ESPGHAN recently increased its suggested range to 3–8 mEq/kg/day, whereas the AAP maintains more conservative recommendations, which recommends a Na intake of 3–5 mEq/kg/day for clinically stable preterm infants with birth weight <1500 g [15].

This narrative review aims to synthesize current and emerging evidence on sodium requirements and supplementation in preterm neonates, to propose practical clinical strategies, and to highlight the need for further research to refine evidence-based nutritional guidelines.

## 2. Risk Factors for Hyponatremia in Preterm Neonates

Hyponatremia after the first 5 days of life is common in very preterm infants. Early studies demonstrated that negative sodium balance occurred in all infants born <30 weeks’ gestation, in 70% of those born at 30–32 weeks, 46% at 33–35 weeks, and none after 36 weeks [16]. In very-low-birth weight (VLBW) infants, the incidence of hyponatremia (<130 mEq/L) increased from 25% in the first week to 65% thereafter [17,18]. In a retrospective cohort of 126 preterm infants born before 36 weeks of gestation, hyponatremia defined as a sodium level ≤ 132 mEq/L or 133–135 mEq/L with oral sodium supplementation, was found to 29.4% of the infants [19].

Nephrogenesis begins around the fifth week of gestation and continues until approximately 34–36 weeks, after which no new nephrons are formed [20]. Consequently, preterm birth interrupts this developmental process, leading to fewer nephrons at birth and potentially suboptimal renal function that may persist throughout life [21].

In the fetus, sodium homeostasis is maintained through placental transfer and efficient renal conservation. During gestation the fetus accumulates Na at an estimated rate of 1.6–2.1 mEq/kg/day between 27 and 34 weeks of gestation [22]. After birth, preterm infants are generally able to absorb sodium efficiently from the gastrointestinal tract, as they typically lose less than 10% of their intake through fecal excretion [23].

Preterm infants are particularly prone to developing negative sodium balance and hyponatremia due to their limited renal tubular sodium reabsorption and the high sodium losses that occur after birth [3,16,24,25,26]. Extremely preterm neonates (25–30 weeks’ gestation) typically show elevated urinary sodium excretion during the first week of life, which gradually decreases over the following weeks but often remains higher than that of term infants. This increased natriuresis, combined with higher aldosterone levels and reduced tubular responsiveness, contributes to substantial sodium losses and negative sodium balance, especially in very-low-birth weight infants (<1500 g) [3,16,24,25,27]. Conditions common among preterm infants—such as respiratory distress syndrome, patent ductus arteriosus, and exposure to diuretics or other medications—can further worsen sodium wasting. These factors contribute to persistent renal sodium wasting during the first weeks of life [3,28].

One study in preterm infants reported urinary sodium losses of 5.4–7 mEq/kg/day at 2 weeks of age, decreasing to 3.5–4 mEq/kg/day by 8 weeks in infants born between 23 and 29 weeks of gestation. The investigators also noted that urinary sodium losses declined progressively with increasing gestational age and postnatal maturity [29]. Elevated fractional excretion of sodium (FENa) provides evidence of impaired tubular reabsorption. Both FENa and urinary sodium excretion (UNaV) are inversely correlated with gestational age and postnatal age, confirming maturational improvements in renal sodium handling [30,31].

Reported risk factors for hyponatremia include extreme prematurity, birth weight <1000 g, feeding with fortified human milk, and the presence of intraventricular hemorrhage [18,32].

For preterm infants, maternal milk alone does not provide sufficient sodium to support optimal growth. Although breast milk remains the preferred source of nutrition for premature infants [33], its sodium content declines rapidly during the first few days after birth and is influenced by both the method of milk expression and the maternal serum sodium concentration [34,35,36]. Evidence from recent studies confirms that fortified human milk, although nutritionally enhanced, still fails to meet the complete sodium and nutrient requirements of preterm infants [37,38]. The ESPGHAN guidelines similarly emphasize that breast milk, even when fortified, may not provide adequate sodium to meet the needs of this population [36]. Likewise, donor human milk, even when supplemented with commercial fortifiers, is often insufficient to achieve recommended sodium intakes [39].

Moreover, common pharmacologic interventions such administration of diuretics, indomethacin, aminoglycosides (especially Gentamicin) [40,41] and vancomycin further exacerbate renal sodium loss and hyponatremia [42]. Low phosphate levels a common condition in the preterm neonate also leads to increased sodium and calcium tubular excretion as these to ions are associated in the level of proximal tubule [43]. The combination of prematurity with in utero retardation (IUGR) renders the kidney to higher electrolytes loses among which sodium [44]. Beyond these pathophysiological mechanisms, sodium also serves as a critical substrate for growth. Thus, preterm infants not only experience increased sodium losses but also possess elevated sodium requirements to support active cellular growth and tissue development in the early postnatal period. The summary of the risk factors for hyponatremia in preterm infants is shown in Table 1.

## 3. Hyponatremia and Growth Failure

Chronic total body sodium depletion has been shown to adversely affect somatic growth in both infants and children [45,46]. Infants with low urinary sodium (<10 mmol/L) show a decline in weight of ~0.2 Z-score per week, whereas sodium-replete infants demonstrate positive weight gain. In parallel, higher early postnatal growth velocity in extremely preterm infants (≈21 vs. 12 g/kg/day) is associated with substantially lower rates of cerebral palsy (6% vs. 21%) and neurodevelopmental impairment (29% vs. 55%) at 18–22 months, supporting a quantitative link between sodium-related growth failure and adverse neurodevelopment [47,48]. The mechanisms linking sodium deficiency to growth impairment are not yet fully elucidated. One of the earliest described pathways is the development of intracellular acidosis [49]. When extracellular sodium levels fall, the activity of the sodium–hydrogen exchanger (NHE1) diminishes, reducing hydrogen ion extrusion from the cell. The resulting intracellular acidification disrupts normal cell metabolism, inhibits DNA and protein synthesis, and impairs the function of growth factor receptors. Experimental models demonstrate that intracellular acidosis reduces chondrocyte proliferation and impairs anabolic activity in multiple tissues, mechanisms that plausibly contribute to poor linear and somatic growth in sodium depleted preterm infants [49].

A second major mechanism involves impaired intestinal nutrient absorption, particularly glucose transport via the sodium–glucose cotransporter (SGLT-1). SGLT-1 relies on a favorable extracellular sodium gradient to drive active uptake of glucose across the apical membrane of enterocytes [50]. In states of sodium depletion, the reduced sodium gradient leads to diminished glucose absorption. This leads to reduced caloric availability, as less glucose is transferred from the gut into the bloodstream, and reduced enteroendocrine stimulation, since glucose delivery to the distal intestine is a key trigger for secretion of trophic hormones such as GLP-1 and other growth-promoting peptides. The combined effect is impaired nutrient utilization and reduced stimulation of growth pathways. Sodium deficiency also impairs several Na^+^-dependent amino acid transporters, limiting amino-acid uptake and protein synthesis. Additionally, inadequate sodium intake disrupts cellular volume regulation, a known signal for anabolic pathways: cell shrinkage inhibits mTOR signaling and reduces growth rate. Preterm infants, who already have high anabolic demands, are therefore particularly vulnerable [9,50]. Collectively, these mechanisms underscore the critical role of sodium in nutrient absorption, metabolic regulation, and overall growth homeostasis in the preterm infant.

## 4. Sodium Assessment and Supplementation in Preterm Neonates

Preventing sodium imbalance in preterm infants requires careful management of both fluid and sodium intake [4]. Sodium intake in preterm infants is from a combination of parenteral and enteral sources. The American Academy of Pediatrics currently recommends a Na intake of 3–5 mEq/kg/day for clinically stable preterm infants with birth weight <1500 g [15]. However, accumulating evidence indicates that many preterm infants require higher sodium intakes to compensate for substantial urinary sodium losses [29,51,52]. Reflecting these findings, the European Society for Paediatric Gastroenterology, Hepatology and Nutrition (ESPGHAN) Committee on Nutrition has updated its guidance, recommending 3–8 mEq/kg/day in infants receiving high energy and protein intakes or experiencing notable sodium losses [36].

Serum sodium concentration is often used as a clinical indication for initiating sodium supplementation in neonates. However, serum sodium primarily reflects the balance between sodium and water rather than the absolute total body sodium content. Evidence from in vivo studies indicates that serum sodium is a relatively poor indicator of overall sodium status [53,54]. In contrast, urinary sodium concentration provides more useful information for assessing sodium depletion and guiding supplementation strategies [12]. In preterm infants, a low urinary sodium concentration (<30–40 mEq/L) generally suggests, though does not definitively confirm, depletion of total body sodium stores [55]. Therefore, routine urine sodium monitoring may allow earlier and more precise correction of sodium deficits than reliance on serum levels alone.

A 2023 meta-analysis including nine studies of preterm infants born before 37 weeks’ gestation or with birth weight <2500 g found that higher late sodium intake reduced the incidence of hyponatremia (<130 mEq/L). However, only one small study reported that increased late sodium intake was associated with a lower incidence of postnatal growth failure [4].

Across more than a dozen studies spanning several decades [13,29,51,56,57,58,59,60,61,62,63,64], the evidence consistently demonstrates that sodium supplementation in preterm infants—particularly those born very preterm or with very low birth weight—is associated with improved growth outcomes without significant safety concerns. The studies conducted regarding sodium supplementation in preterm infants are shown in Table 2. Earlier studies have shown that very preterm neonates fail to achieve adequate weight gain when sodium supplementation is not given [16,60]. In contrast, enhanced enteral sodium intake has been shown to promote improved growth outcomes [13,29,51,59,64]. While evidence supports sodium supplementation for biochemical stability, data on its long-term growth benefits remain limited and warrant further trials.

Randomized controlled trials, including recent high-quality modern studies, demonstrate that sodium intakes of approximately 3–5 mEq/kg/day improve weight gain velocity, reduce postnatal growth failure, and support better early fluid balance. In a randomized controlled trial, Isemann et al. supplemented preterm infants born at <32 weeks’ gestation with 4 mEq/kg/day of sodium. Infants in the intervention group had fewer instances of serum sodium <135 mEq/L and demonstrated greater weight gain velocity during the first six weeks of life, though length and head circumference growth did not differ significantly between groups [51].

Studies using urine sodium–based algorithms (Segar et al.; Stalter et al.) show that structured, physiology-driven protocols can identify infants with sodium deficiency early and safely increase sodium intake [13,29]. Segar et al. supplemented preterm infants with 4 mEq/kg/day above standard sodium intake, increasing supplementation by an additional 2 mEq/kg/day if urine sodium remained below target. Urinary sodium was assessed every two weeks beginning at two weeks of age. Infants receiving supplementation showed greater mean weight and weight gain at both 2 and 8 weeks compared with controls [29]. A similar protocol was followed by Stalter et al. in which sodium supplementation was initiated when spot urine sodium was below the expected range for gestational and postnatal age or when serum sodium ≤132 mEq/L from two weeks postnatal age. Infants <30 weeks’ gestation received 4 mEq/kg/day of sodium above dietary intake, with weekly adjustments for growth. Among infants born at 26–29 weeks’ gestation, supplementation was associated with a higher mean body weight z-score at 8 weeks, greater head circumference z-score at 16 weeks, and reduced time on mechanical ventilation, while no growth effects were observed in infants ≥30 weeks’ gestation [13].

Long-term neurodevelopmental outcomes have not been systematically reported in most studies evaluating sodium supplementation in preterm infants. Notably, the 2023 meta-analysis found no data assessing the relationship between sodium supplementation and long-term neurodevelopmental outcomes. In an early study by Al-Dahhan et al., preterm infants receiving restricted sodium intake had poorer neurodevelopmental outcomes at 10–13 years of age compared with sodium-supplemented infants [65]. Additionally, hyponatremia has been identified as a risk factor for sensorineural hearing loss [66] and cerebral palsy [67]. Although recent studies demonstrate improved early weight gain with sodium supplementation, long-term neurodevelopmental outcomes were not assessed, and a causal relationship between serum sodium fluctuations and neurodevelopment remains unproven.

A double-blind randomized controlled trial enrolled neonates born between 25 and 30+6 weeks and provided Na supplementation of 4 mEq/kg/day orally. The authors concluded that early postnatal sodium supplementation significantly enhances in-hospital weight and linear growth, with untreated infants requiring an additional eight days to achieve equivalent monthly weight gain [63]. Petersen et al. reported that sodium-supplemented infants were smaller and less mature at baseline but exhibited improved serum sodium levels, weight gain, and head circumference growth without an increase in hypernatremia [64]. These findings highlight early sodium supplementation as a practice guided by gestational age and urinary sodium, which appears both safe and effective in promoting growth among the most immature infants.

Our experience at our center has been to use the upper limit of the earlier guideline recommendations, namely 4–5 mEq/kg/day, with weekly assessments of fractional excretion of sodium (FENa), and more frequent monitoring when clinically indicated [40]. Sodium supplementation was subsequently individualized based on these results that usually dictated an up titration of sodium supplementation. From our clinical experience, we observed that hyponatremia was not rare even at this level of Na supplementation while, hypernatremia occurred only extremely rarely.

Based on the available data [13,29,36] and on our practice an approach to sodium supplementation in preterm infants could be the following as shown in Algorithm 1.
**Algorithm 1.** Algorithm for Sodium Supplementation in Preterm Infants based on available studies [13,29,36].**Step 1.** Suspect sodium deficiency in preterm infants especially if gestational age < 30–32 weeks and/or birth weight < 1500 g and postnatal age > 5–7 days**Step 2**. Start with sodium intake consistent with gestational age and clinical stability**Step 3.** Measure spot urine sodium and serum sodium concentration at 1 week of postnatal age Urine sodium below expected values for gestational and postnatal age or serum Na ≤ 132 mEq/L suggest sodium deficiencyValues should not be obtained within 48 h of use of diuretic agent**Step 4.** If urine sodium is below target or serum Na ≤ 132 mEq/L, increase sodium intake by 4 mEq/kg/day above current intake**Step 5.** Recheck urine sodium every 1–2 weeks and monitor for weight gain velocity and weight Z-score, serum sodium, chloride, potassium, acid–base status and clinical statusif urine sodium remains below target or growth remains suboptimal increase supplementation by an additional 2 mEq/kg/dayif urine sodium is within target range and growth is appropriate maintain current sodium intakemonitor for weight gain velocity and weight Z-scoremonitor for serum sodium, chloride, potassium, acid–base statusmonitor for clinical status and adverse events such as systemic hypertension, bronchopulmonary dysplasia, culture positive sepsis, and necrotizing enterocolitis**Step 6.** Continue ongoing monitoring and safety regarding serum sodium, excessive chloride load, hypertension or fluid retention.Continue supplementation unless serum Na >144 mEq/LConsider discontinuation of supplementation at 38 weeks’ postmenstrual age

## 5. Complications from Na Supplementation

Rapid fluctuations in serum sodium concentration during the first month of life have been associated with adverse long-term neurological outcomes [17]. Potential complications of excessive sodium supplementation include hypernatremia, which may lead to fluid retention, hypertension, respiratory compromise, intraventricular hemorrhage, seizures, and thrombosis [68]. However it should be noted that no infant required sodium supplementation to be discontinued due to hypernatremia (serum sodium concentration > 144 mEq/L) [29]. Prolonged exposure to higher sodium intakes may theoretically increase renal workload and contribute to long-term renal or cardiovascular risk. However, such adverse outcomes have not been demonstrated in the available neonatal studies to date. These potential risks warrant evaluation in well-designed, long-term prospective studies.

Concerns have been raised regarding the risk of hyperosmolar feeds contributing to necrotizing enterocolitis (NEC); however, in studies sodium supplementation has not been associated with feed intolerance, NEC, hyponatremia, or hypernatremia or an increase in common prematurity-related morbidities [13,51,62,63,69,70]. Sodium is often administered as sodium chloride, which can contribute to a higher chloride load and, in some clinical settings, has been associated with hyperchloremic metabolic acidosis when chloride intake exceeds bicarbonate buffering capacity [71,72]. Furthermore, alterations in sodium handling and increased sodium delivery to the distal nephron, such as with diuretic use, stimulate sodium reabsorption in exchange for potassium and hydrogen ion secretion, which can increase renal potassium excretion and affect acid–base balance [73]. Although, no study reported these complications such as hyperchloremic metabolic acidosis or increased potassium excretion in the preterm infants supplemented with Na, clinicians should remain vigilant for these potential physiological disturbances and monitor infants closely.

Overall, current evidence supports the safety of sodium supplementation when dosing and monitoring are carefully adjusted to the infant’s gestational age and renal function

## 6. Conclusions and Future Directions

Despite decades of investigation, optimal sodium management in preterm infants remains an evolving field. Current evidence clearly demonstrates that immature renal function, high urinary sodium losses, and insufficient sodium content in human milk collectively place very preterm infants at substantial risk for negative sodium balance, hyponatremia, and postnatal growth failure.

Although some early studies reported neutral effects of sodium supplementation on growth, these typically involved lower doses or short intervention periods [58]. Importantly, no study has demonstrated adverse growth or clinical outcomes attributable to sodium supplementation when appropriately dosed and monitored (Table 2). In contrast, hyponatremia has consistently been associated with impaired growth and adverse neurodevelopmental outcomes [45,47,65]. Variability in reported effects likely reflects differences in study design, gestational age, and reliance on serum rather than physiological markers of sodium status. More recent studies using individualized, urine sodium–guided approaches consistently report improved growth without increased morbidity, supporting the safety of targeted sodium supplementation in very preterm infants [13,29].

Although both observational studies and randomized trials consistently show that sodium supplementation improves biochemical stability and supports early growth, several important gaps remain. First, the physiological variability in sodium handling among preterm infants of different gestational ages suggests that a “one-size-fits-all” supplementation strategy may be inadequate. Emerging data support the use of individualized, physiology-based approaches, particularly algorithms incorporating urine sodium concentrations to guide supplementation. However, these strategies have not yet been validated in large, multicenter prospective trials. A major future priority is determining whether routine, longitudinal urine sodium monitoring improves growth, reduces morbidity, or affects long-term neurodevelopment compared with traditional serum-based approaches.

Second, although short-term growth outcomes appear to benefit from higher sodium intake, there is limited evidence on long-term growth, cardiovascular, and neurodevelopmental effects. This is particularly relevant given concerns about potential risks of excessive supplementation, including hypertension or abnormal fluid balance, despite current studies showing reassuring safety profiles. Future research should clarify the upper safe limit of sodium intake in extremely preterm infants and assess whether early sodium supplementation has long-term beneficial effects. Moreover, large, prospective, multicenter randomized trials are needed to evaluate the risk of NEC.

Third, nutritional practices have evolved considerably, including increased use of donor human milk and standardized fortifiers. As these feeding strategies inherently provide lower sodium than maternal milk, reevaluation of sodium recommendations in the context of contemporary nutrition is urgently needed. Studies should also explore how sodium interacts with other growth-limiting factors, such as protein intake, phosphate deficiency, diuretic exposure, and intrauterine growth restriction, to influence growth and metabolic outcomes.

Finally, research is needed to further elucidate how sodium depletion affects cellular growth pathways, intestinal function, microbiome development, and brain maturation. Understanding these mechanisms may help refine optimal sodium targets and identify infants who may benefit most from supplementation. A multicenter randomized controlled trial comparing sodium supplementation strategies guided by urinary sodium versus serum sodium levels, with long-term follow-up of growth and neurodevelopment, is essential to address current knowledge gaps and inform clinical practice.

In summary, while current evidence supports the safety and efficacy of sodium supplementation in very preterm infants, substantial opportunities remain to refine assessment methods, personalize supplementation strategies, and evaluate long-term outcomes. Future studies should focus on integrating physiological markers with modern nutritional practices to develop clear, evidence-based guidelines that optimize sodium balance, support healthy growth, and improve long-term development in this highly vulnerable population.

## Figures and Tables

**Table 1:** Risk factors for hyponatremia in preterm neonates.

Risk Factor	Comment
Prematurity	Immature renal tubular function and low glomerular filtration rate Poor ability to conserve sodium and excessive urinary sodium loss
Inadequate Na intake	Exclusive feeding with unfortified human milk Donor human milk or fortified breast milk still insufficient in sodium content Prolonged parenteral nutrition without adequate sodium supplementation
Fluid overload or excessive free water administration	Dilutional hyponatremia
Intraventricular hemorrhage	Associated with SIADH and altered sodium regulation
Sepsis, respiratory distress syndrome, or asphyxia	Increased ADH secretion
Necrotizing enterocolitis	Sodium loss through the gastrointestinal tract
Medications or other metabolic disturbances (diuretics, indomethacin, aminoglycosides, vancomycin, low serum phosphate)	Increased renal sodium excretion Decreased renal blood flow and altered sodium handling

ADH: Antidiuretic Hormone, SIADH: Syndrome of Inappropriate Antidiuretic Hormone secretion.

**Table 2:** Sodium Supplementation Studies in Preterm Infants shown in chronological order [3,13,16,51,57,58,59,60,61,62,63,64].

Study	Study Type	GA (Weeks) or Birth Weight (g)	Population (Supplemented vs. Controls)	Sodium Evaluation	Sodium Supplementation (mEq/kg/day)	Impact on Weight/Growth on Supplemented Neonates	Complications
Al-Dahhan et al., 1984 [56]	Observational	27–34	22 vs. 24	Urine, serum, stool	4–5 (day 4–14)	Less weight loss; earlier regain	None
Ekblad et al., 1987 [57]	RCT	<34	10 vs. 10	Serum	4	Similar early weight loss	None
Shaffer et al., 1989 [58]	RCT	700–1500 g	20	Serum	3 vs. 1	No weight difference	None
Ayisi et al., 1992 [59]	RCT	1001–1500 g	41 vs. 25	Serum, urine	3	Increased weight, length, head circumference growth	None
Vanpee et al., 1995 [60]	RCT	29–34	10 vs. 10	Urine	4 (day 4–14)	Less weight loss	None
Hartnoll et al., 2000 [61]	RCT	25–30	24 early vs. 22 delayed	Serum	4 early vs. delayed	No difference in birth weight regain	Early supplementation increased oxygen need
Isemann et al., 2016 [51]	RCT	<32	27 vs. 26	—	4 (day 7–35)	Decreased growth failure	None
Segar et al., 2018 [29]	Observational (historical cohort)	26–29	40 vs. 50	Urine	4; Additionally 2 if low urine Na	Increased weight Z-score	None
Sanchez et al., 2023 [62]	RCT	<35	19 vs. 23	Serum	5	Less weight loss	None
Petersen et al., 2024 [64]	Observational	22–32	821	Serum, urine	Variable	Increased weight and head circumference growth	None
Amiti et al., 2025 [63]	Double-blind RCT	25–30	52 vs. 52	Serum	4	Increased weight gain velocity	None
Stalter et al., 2025 [13]	Retrospective cohort	26–33	26–29 wks: 225 vs. 157 30–33 wks: 157 vs. 153	Urine	4; Additionally 2 if low	Increased weight Z-score (26–29 wks) No impact on growth (30–33 wks)	None

GA: gestational age, NEC: necrotizing enterocolitis, RCT: randomized controlled trial, VLBW: very low birth weight, wks: weeks.

## Data Availability

No new data were created or analyzed in this study.

## Abbreviations

The following abbreviations are used in this manuscript:

Na	Sodium
ADH	Antidiuretic Hormone
SIADH	Syndrome of Inappropriate Antidiuretic Hormone secretion
VLBW	Very low birth weight
